# Identification of novel candidate genes and predicted miRNAs in atopic dermatitis patients by bioinformatic methods

**DOI:** 10.1038/s41598-022-26689-8

**Published:** 2022-12-21

**Authors:** LiangHong Chen, Xin Qi, JingYu Wang, JiaLi Yin, PeiHong Sun, Yan Sun, Yan Wu, Li Zhang, XingHua Gao

**Affiliations:** 1 Department of Dermatology, The First Hospital of China Medical University, 155 North Nanjing Street, Shenyang, 110001 China; 2 Department of Emergency Medicine, Shengjing Hospital of China Medical University, Shenyang, China; 3 Key Laboratory of Immunodermatology, Ministry of Education and NHC, National Joint Engineering Research Center for Theranostics of Immunological Skin Diseases, Shenyang, China

**Keywords:** Computational biology and bioinformatics, Genetics, Immunology, Biomarkers, Diseases

## Abstract

Atopic dermatitis (AD) is a common, chronic inflammatory dermatosis with relapsing eruptions. Our study used bioinformatics to find novel candidate differentially expressed genes (DEGs) and predicted miRNAs between AD patients and healthy controls. The Mesh term “atopic dermatitis” was retrieved to obtain DEGs in GEO datasets. DEGs between AD patients and healthy controls were analyzed using GEO2R. Overlapping DEGs between different datasets were obtained with use of Draw Venn software. GO and KEGG enrichment analyses were conducted by the use of DAVID. STRING and miRWalk were used to individually analyze PPI networks, interactions of candidate genes and predicted miRNAs. A total of 571 skin samples, as retrieved from 9 databases were assessed. There were 225 overlapping DEGs between lesioned skin samples of AD patients and that of healthy controls. Nineteen nodes and 160 edges were found in the largest PPI cluster, consisting of 17 up-regulated and 2 down-regulated nodes. Two KEGG pathways were identified, including the cell cycle (CCNB1, CHEK1, BUB1B, MCM5) and p53 (CCNB1, CHEK1, GTSE1) pathways. There were 56 nodes and 100 edges obtained in the miRNA-target gene network, with has-miR-17-5p targeted to 4 genes and has-miR-106b-5p targeted to 3 genes. While these findings will require further verification as achieved with experiments involving in vivo and in vitro modles*,* these results provided some initial insights into dysfunctional inflammatory and immune responses associated with AD. Such information offers the potential to develop novel therapeutic targets for use in preventing and treating AD.

## Introduction

As a common, chronic inflammatory dermatosis, atopic dermatitis (AD) is characterized by relapsing eczematous and pruritic skin lesions^1^. The prevalence rate of AD ranged from 1 to 20% worldwide^2^, and the incidence of this condition has been gradually increasing. This disease persists throughout the life of these patients, from infantile to adults stages, and greatly impacts their daily lives, as well as social and leisure activities^3^. In China, as determined in 2019, AD has been reported to be the 24th most burdensome of the 369 most commonly identified diseases and is a notable source of public health concern^4^. In children, irritant dermatitis and food allergies are typical aggravating conditions, while sweating and psychological stress significantly impact AD occurrence in adults^3^.

Skin barrier dysfunction, Th2 cell-mediated immunity, microbial colonization, oxidative stress, as well as genetic and environmental factors have all been reported as contributing to the pathogenesis of AD^5–8^. A total of 46 genes and 34 genetic loci have been reported to be related to sporadic AD worldwide^9^. As one of the most notable variant genes in AD, filaggrin (FLG) plays a vital role in epidermal terminal differentiation and skin barrier homeostasis^10,11^. Microbial colonization is also involved in the etiology of AD, with increased expressions of interleukin (IL)-4/13/17/22/31, as well as other cytokines^12^, and thus enhanced Th2-mediated inflammation, being associated with AD^13^. AD is always accompanied with asthma, eosinophilic esophagitis, urticaria and rhinitis. In very young children (0–2 years of age), prevalence rates of the above type 2 inflammatory diseases listed above were twice that as observed in teenagers and adults, with this incidence decreasing as a function of the severity of the eruptions^14^. Therefore, AD is considered as a systemic disease.

Traditionally, treatment of AD has consisted of systemic drugs (cyclosporine, methotrexate, azathioprine, and mycophenolate mofetil), topical ointments (corticosteroids, calcineurin inhibitors) and phototherapies (NB-UVB, UVA1, PUVA)^15,16^. More recently, administrations of more specific biological agents, e.g. IL-4/13 receptor blocker (dupilumab), phosphodiesterase V inhibitor (crisaborole) and Janus kinase inhibitors (ruxolitinib, tofacitinib, baricitinib, abrocitinib, upadacitinib) have shown vital roles in AD treatment^17^. However, AD remains difficult to treat and frequently recurs. Therefore, alternative effective therapies that can limit recurrence rates are urgently needed.

In the present study, the microarray data of AD patients were searched from the database of Gene Expression Omnibus (GEO), and some novel candidate differentially expressed genes (DEGs) and predicted miRNAs in lesioned versus non-lesioned skin samples of AD patients as well as that of healthy controls were identified using bioinformatics. Our goal with this analysis was to establish a theoretical basis for some more precise therapies to treat AD.

## Materials and methods

### Microarray data

The Mesh term “atopic dermatitis” was retrieved to obtain DEGs in GEO datasets (a public database of the National Center of Biotechnology Information program, http://www.ncbi.nlm.nih.gov/geo) on Aug 24, 2021. Datasets from the same platform consisting of skin samples from mild to moderate adult AD patients were enrolled and then downloaded. Datasets without DEGs or those with adjustments of *P* < 0.05 were excluded. After screening, 9 microarray datasets, consisting of GSE16161, GSE32924, GSE59294, GSE99802, GSE107361, GSE120899, GSE130588, GSE133385 and GSE133477, were included in our study,. The platform of the 9 datasets was based on GPL570 (Affymetrix Human Genome U133 Plus 2.0 Array, Affymetrix Inc., Santa Clara, CA, USA).

### Identification of DEGs

To identify novel candidate genes, DEGs between lesioned/non-lesioned skin samples of AD patients and those of healthy controls were assessed using GEO2R. Genes without gene names and duplicates were exclude. The fold change (FC) was used as a means to show relative differences in the expressions of these genes. Genes with log_2_FC > 0 represented up-regulated genes, while those of log_2_FC < 0 indicated down-regulated ones. Genes with |log_2_FC|> 1 were included for further analysis. Adjusted *P* < 0.05 was considered as indicating statistically significant differences. Overlapping DEGs among different datasets were conducted by use of Draw Venn software (http://bioinformatics.psb.ugent.be/webtools/Venn/).

### Gene ontology (GO) and Kyoto Encyclopedia of Genes and Genomes (KEGG) enrichment analysis

GO enrichment analysis was applied to identify gene functions and KEGG enrichment analysis was conducted to generate related signaling pathways of candidate DEGs between lesioned vesus non-lesioned skin samples of AD patients and those of skin samples from healthy controls. The analyses were performed using an online functional annotation tool, Database for Annotation, Visualization, and Integrated Discovery (DAVID) Bioinformatics Resources 6.8 (http://david.abcc.ncifcrf.gov/). For the GO analysis, homo sapiens, official-gene-symbol and gene list were set as species, identifier and list type, respectively. Biological process (BP), cell component (CC) and molecular function (MF) evaluations were then conducted, with a cutoff value of *P* < 0.05. Bubble plots were generated using the Ggplot2 package of R software version 3.6.3.

### Protein–protein interaction (PPI) network analysis

The search Tool for the Retrieval of Interacting Genes/proteins (STRING) was performed to analyze PPI networks between lesioned/non-lesioned skin samples of AD patients and those of healthy controls (https://string-db.org). DEGs, as restricted to homo sapiens, were uploaded to “multiple proteins by names/identifiers”. Network nodes represented proteins with different node colors indicating proteins with different functions. Empty nodes indicated proteins with unknown 3D structures while filled nodes indicated those with known or predicted 3D structure. Edges represented protein–protein associations, with different colors signifying different interactions (known, predicted or others). The greater the number of edges, the greater the connections present between different proteins. Potential correlations between DEGs were further evaluated using Cytoscape (Version 3.8.2)^18^. Modulations of PPI networks were conducted with use of the plug-in unit, MCODE (Version 2.0.0) in Cytoscape^19^. Clusters were found in whole networks, with cutoffs for degree = 2, haircut, node density cutoff = 0.1, k-core = 2, node score cutoff = 0.2, and maximal Depth = 100. Clusters with values ≥ 3 nodes were selected.

### Gene-miRNA analysis

Interactions between candidate genes and predicted miRNAs in lesioned/non-lesioned skin samples of AD patients and those of healthy controls were conducted using miRWalk 3.0 (http://mirwalk.umm.uni-heidelberg.de/). The parameters in TargetScan, miRDB and miRTarBase consisted of a relationship score > 0.95 and a target gene binding region = 3′UTR. Results were visualized using Cytoscape 3.8.2^20,21^.

## Results

**Figure 1:**
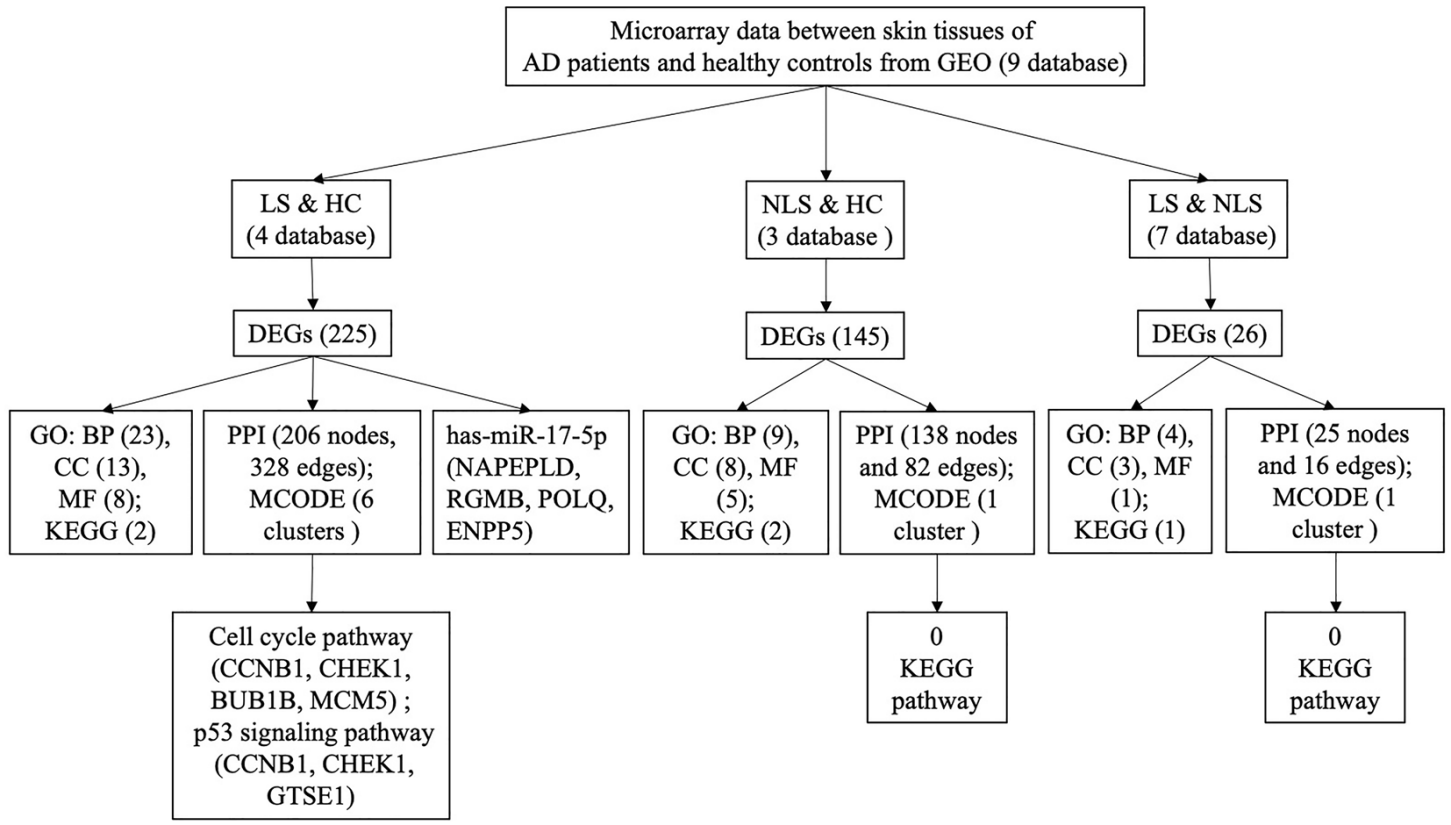
Flow diagram of the analysis strategy. *DEG* differentially expressed gene, *GO* gene ontology, *BP* biological process, *CC* cell component, *MF* molecular function, *KEGG* Kyoto Encyclopedia of Genes and Genomes, *PPI* protein–protein interaction, *LS* lesioned skin samples of AD patients, *HC* healthy controls, *NLS* non-lesioned skin samples of AD patients.

A flowchart of the analysis strategy is shown in Fig. 1.

### DEGs in AD patients versus healthy controls

A total of 571 skin samples were enrolled from 9 databases and included 298 samples from lesioned and 225 from non-lesioned skin samples of AD patients along with 48 samples from healthy controls.

**Figure 2:**
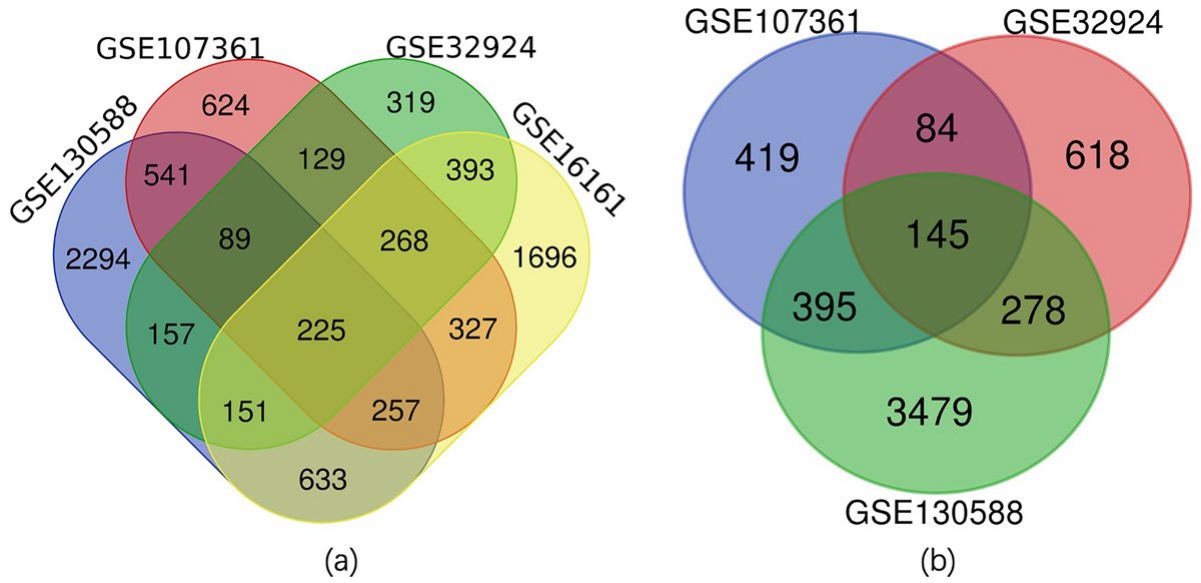
Overlapping DEGs obtained among the different groups as determined using Draw Venn software. (**a**) lesioned skin samples of AD patients versus healthy controls; (**b**) non-lesioned skin samples of AD patients versus healthy controls.

DEGs found between lesioned skin samples of AD patients and those of healthy controls were identified within 4 databases. There were 3950, 1731, 2460 and 4347 DEGs in GSE16161, GSE32924, GSE107361 and GSE130588, respectively. A total of 225 overlapping DEGs were generated from the 4 databases as revealed using Draw Venn software (Fig. 2a, Table 1).

**Table 1:** DEGs between AD patients and healthy control.

Groups	DEGs
Lesioned skin of AD patients & healthy controls	*CCNB1, AQP9, SPHAR///RAB4A, GEN1, ALDOC, IL1F10, SDC2, SLITRK4, ACADL, JADE1, APOBEC3A_B///APOBEC3A, SLC46A2, CRABP1, IL17D, OAS1, EHBP1L1, LGR5, XAF1, WIF1, MCM5, SPTLC3, SOCS3, LCE1E, RERGL, HBA2///HBA1, ALOX12, ABHD5, CCL18, PNPLA3, GIPC2, FAM171B, OCLN, GALNT6, LOC101930416///LOC101929792///LOC100996724///PDE4DIP, LEPROT///LEPR, CNTN1, PALMD, RGS22, MIR155///MIR155HG, KRBOX1, SYBU, DDX17, ZNF567, SNHG3///SNORA73A, FAXDC2, ANKRD33B, CPE, CALD1, NRBF2, TPR, SERPINA5, PSD3, LOC643923///ELMOD1, GPHN, DEPDC1B, CHEK1, KIF18B, VSNL1, CCL17, CNGA1, ANXA9, MAP4K1, SATB2, ABCC3, HOXA10, SLAMF8, ANG, LOC105379178, IGFL2, CPQ, POLQ, SLITRK6, OASL, TSPAN5, PRR11, PRNP, LINC00302, ACOT1///ACOT2, PGRMC1, ANKFN1, ENPP5, PSAT1, PTPN21, GPR34, ADGRL3, KLK5, NMNAT3, DGAT2, ISG20, NFATC2IP, ITFG1, PSORS1C2, COBL, FOXD1, HSD11B1, S100A7A, SGO2, ADGRV1, ADGRF4, SERPINB12, ATRN, S100A9, BIRC3, FIBIN, CYP39A1, SUSD2, GLT8D2, KPNA2, SERPINB9, MSMB, TMEM99, NBR1, HLF, APOBEC3B, C15orf59, PCDHB9///PCDHB10, AURKA, NDNF, VIT, TRIL, C5orf46, SVIP, MAP7, ETV7, HS3ST6, IL13RA1, C1QTNF7, HPGDS, SPRR4, BMP2, FAM107B, IRS2, BCL6, GTSE1, NUF2, SSBP2, MMP12, CHMP4C, ANGPTL1, RGS1, PSG7, PCSK1N, ECT2, SLC1A6, ALOXE3, CENPE, CTNNBIP1, LINS1, RNASE7, CEP55, DTYMK, MACC1, GLRX, MKI67, GDE1, LOX, CFLAR, CD47, GFRA1, COL8A2, PSAPL1, KIAA0101, ACOT8, RANBP9, LOC143286, ACER1, RGMB, MPZL2, FABP7, AGR3, NAPSB, APOL6, CARD18, TSPAN8, CAMK2D, CTSV, MIR15A///DLEU2L///DLEU2, ERAP1, MSH5-SAPCD1///SAPCD1, TMEM246, PGM5-AS1, FBLN1, AKTIP, CLDN23, LPCAT2, TNMD, CYP7B1, DEFB4B///DEFB4A, MIR21///VMP1, AGTR1, NAPEPLD, CD3D, BCOR, SH3TC1, SGCG, EPHX2, MED23, ASPM, TLE1, PRSS12, METTL9, BTC, IL37, TRIM13, STXBP6, ZC3H12D, ZDHHC9, SERPINB4, STK4, DLX5, ITM2A, BUB1B, HMMR, ZBED2, HLA-DQB2, ABLIM3, TMEM254, PTGS1, PARD3, CYP2J2, NCAPG, COL6A6, C1orf68, BCKDHB, CRCT1*
Non-lesioned skin of AD patients & healthy controls	*CD44, GPR78///CPZ, AQP9, TMEM19, IL1F10, TTLL4, ZNF227, ASAH1, SORBS2, SLC46A2, ARHGEF26, TANK, SQLE, WIF1, SPTLC3, LCE1E, USP38, CITED2, ATP2C2, OCLN, RNF146, LEPROT///LEPR, PALMD, DIO2, PSMD10, MTPN, DDX17, ATP6AP2, ITCH, CPE, CALD1, NRBF2, ODF3B, CCDC126, RNASE4, HDLBP, LOC643923///ELMOD1, GPHN, LAMP1, RFWD2, SLC25A1, PDXK, ORMDL3, ANXA9, LYRM5, MLLT3, IGFL2, CPQ, TSPAN5, PRNP, LINC00302, ANKFN1, CYP2C18, PTPN21, GPR34, KLK5, UBE4B, PSORS1C2, GAS7, FBXO45, LPAR1, ADGRF4, GATM, SERPINB12, ATRN, FIBIN, CYP39A1, OGDH, MSMB, MSH6, PLD1, C15orf59, C5orf46, MAP7, ETV7, HPGDS, SPRR4, BMP2, ZBED6, AGA, LSAMP, PSG7, SLC1A6, UBASH3B, ZCCHC6, ALOXE3, NAMPT, LINS1, RNASE7, ABCA9, MKI67, NAV3, GDE1, SDR9C7, CFLAR, SP8, COL8A2, APC, PSAPL1, ITIH4, RANBP9, LOC143286, ACER1, CDYL, AGR3, TMEM86A, AADAC, MPP7, CARD18, CAMK2D, MSH5-SAPCD1///SAPCD1, RHOA, TMEM246, FBLN1, LUC7L, AKTIP, CLDN23, LPCAT2, ARG1, AGTR1, CMTM6, SGCG, M6PR, TPD52, METTL9, BTC, IL37, KLK13, ZDHHC9, PPIF, DLX5, MED28, REL, LRRC28, BLOC1S6, TMEM254, PTGS1, LOC100507472///PCSK6, C3orf38, CYP2J2, NCAPG, JCHAIN, C1orf68, CRCT1, SLC39A6*
Lesioned skin & non-lesioned skins of AD patients	*PHYHIP, C10orf99, TFEC, RGS20, PI15, TMPRSS4, EPSTI1, NR4A3, MMP1, MMP12, HAS3, CXCL2, CTLA4, KRT16, GZMB, SLAMF7, SERPINB3, IGFL1, AKR1B10, SELE, SERPINB4///SERPINB3, OASL, BTC, COL4A4, SERPINB4, FOSL1*

DEGs found between non-lesioned skin samples of AD patients and those of healthy controls were identified within 3 databases. There were 1125, 1043 and 4297 DEGs in GSE32924, GSE107361 and GSE130588, respectively. A total of 145 overlapping DEGs were obtained from the 3 databases (Fig. 2b, Table 1).

DEGs found between lesioned versus non-lesioned skin samples of AD patients were identified within 7 databases. There were 1621, 99, 286, 559, 1039, 946 and 606 DEGs in GSE59294, GSE99802, GSE107361, GSE130588, GSE133385, GSE133477 and GSE140684, respectively. A total of 26 overlapping DEGs were obtained from the 7 databases (Table 1).

### GO and KEGG pathway analysis of DEGs

**Figure 3:**
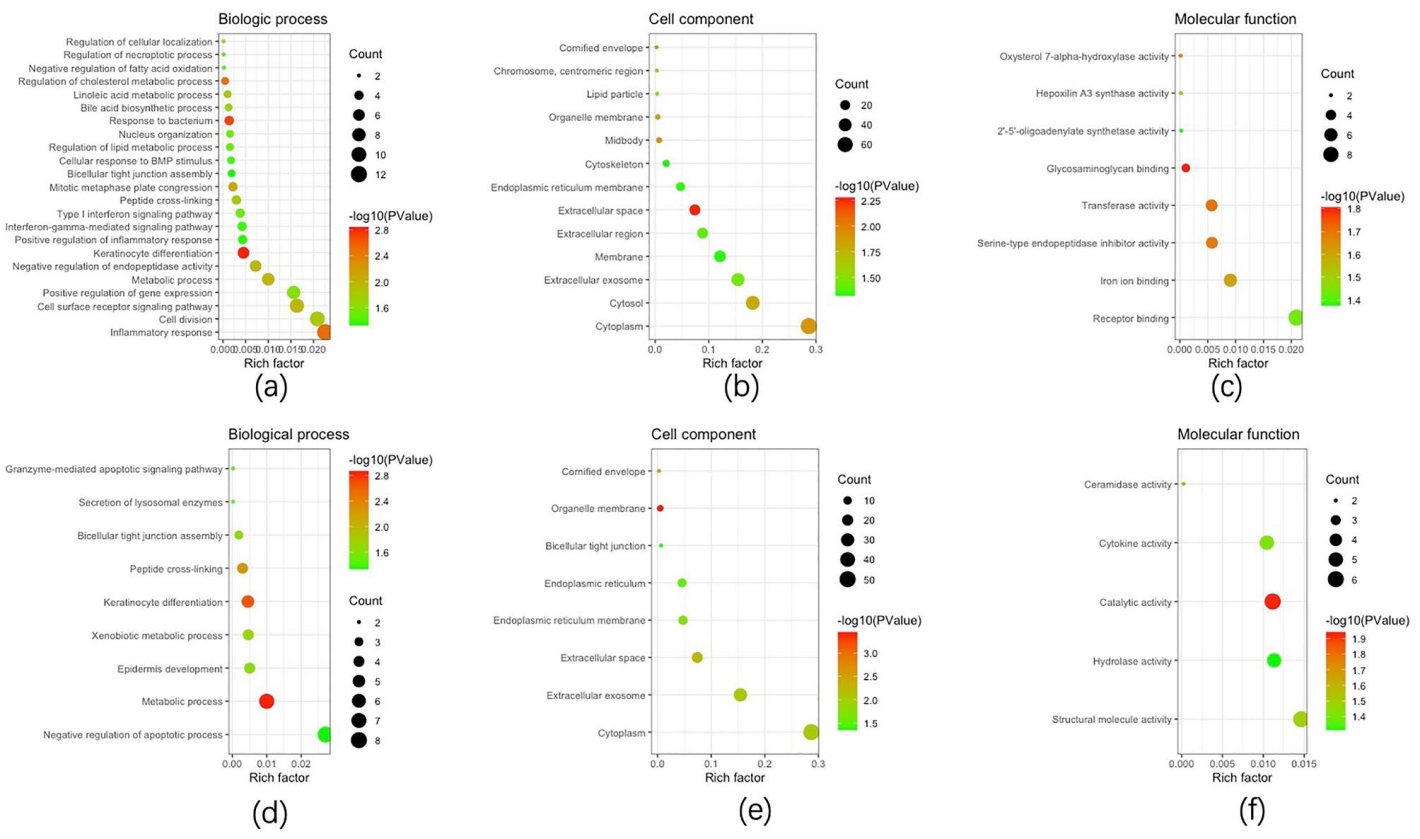
GO analysis of DEGs obtained among the different groups as determined using DAVID. (**a–c**) lesioned skin samples of AD patients versus healthy controls; (**d–f**) non-lesioned skin samples of AD patients versus healthy controls.

Function and KEGG pathway analyses of the DEGs were conducted with the use of GO and KEGG analyses, respectively. The 225 overlapping DEGs of lesioned skin samples from AD patients and those of healthy controls were uploaded to DAVID. There were 23 BP (Fig. 3a), 13 CC (Fig. 3b) and 8 MF (Fig. 3c) GO terms enriched in lesioned skin samples of AD patients (P < 0.05). As compared with samples of healthy controls, 2 KEGG pathways (arachidonic acid metabolism and primary bile acid biosynthesis) were enriched in lesioned skin samples of AD patients (P < 0.05) (Table 1).

The 145 overlapping DEGs from non-lesioned skin samples of AD patients and those of healthy controls were uploaded. There were 9 BP (Fig. 3d), 8 CC (Fig. 3e) and 5 MF (Fig. 3f) GO terms enriched in non-lesioned skin samples of AD patients (P < 0.05). As compared with that of samples from healthy controls, there were 2 KEGG pathways (sphingolipid signaling pathway and lysosome) enriched in non-lesioned skin samples of AD patients (P < 0.05).

The 26 overlapping DEGs from lesioned and non-lesioned skin samples of AD patients were uploaded. Four BP, 3 CC and 1 MF GO terms were identified to be enriched in lesioned skin samples of AD patients (P < 0.05). The DEGs were enriched in the negative regulation of peptidase activity, collagen catabolic process, positive regulation of cell cycle and natural killer cell mediated cytotoxicity for BP, in the proteinaceous extracellular matrix, extracellular space, extracellular region and for CC and in serine-type endopeptidase activity for MF. The amoebiasis KEGG pathway was found to be significantly increased in lesioned skins comparing with non-lesioned skin samples of AD patients (P < 0.05).

### PPI network analysis

**Figure 4:**
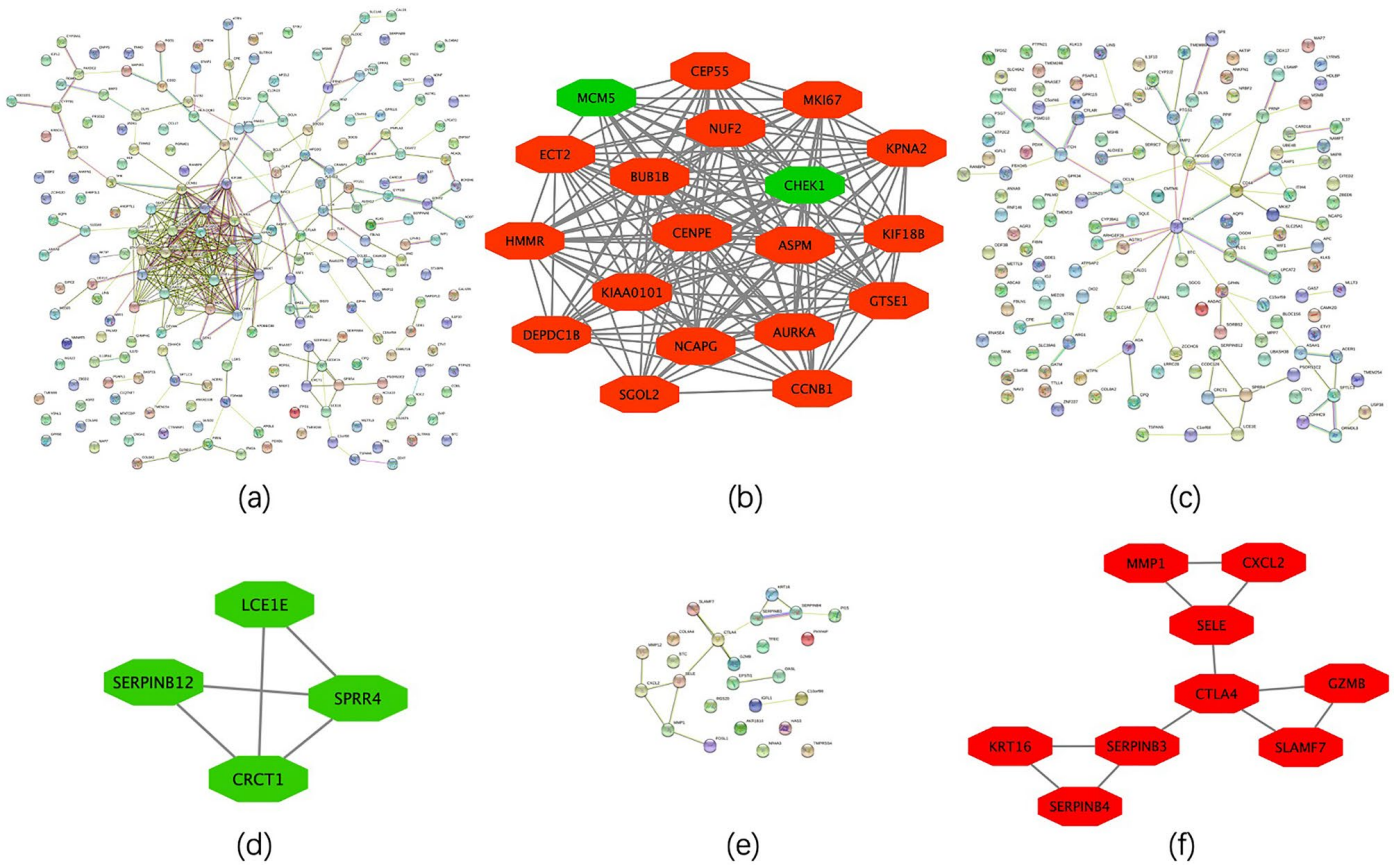
PPI network analysis among the different groups as determined using STRING and Cytoscape (the largest cluster as obtained with MCODE). (**a,b**) lesioned skin samples of AD patients versus healthy controls; (**c,d**) non-lesioned skin samples of AD patients versus healthy controls. (**e,f**) lesioned skin versus non-lesioned skin samples of AD patients.

The overlapping DEGs between different groups were individually uploaded to STRING and then tab separated value files were downloaded and analyzed using MCODE in Cytoscape. For differences between lesioned skin samples of AD patients and those of healthy controls, there were 206 nodes and 328 edges in the PPI network (Fig. 4a), with average node degree = 3.18 and PPI enrichment P-value < 1.0*10^–16^. Six clusters were identified, with 19 nodes and 160 edges in the largest cluster, consisting of 17 up-regulated and 2 down-regulated nodes (Fig. 4b). For differences between non-lesioned skin samples of AD patients and those of healthy controls, the PPI network included 138 nodes and 82 edges (Fig. 4c), with average node degree = 1.19, and PPI enrichment P-value = 0.000289. Only 1 cluster with 4 down-regulated nodes and 5 edges was identified (Fig. 4d). For differences between lesioned versus non-lesioned skin samples of AD patients, the PPI network showed 25 nodes and 16 edges (Fig. 4e), with average node degree = 1.28 and PPI enrichment p-value < 1.47*10^–9^. Only 1 cluster with 9 up-regulated nodes and 11 edges was identified (Fig. 4f).

### KEGG pathway re-analysis

**Figure 5:**
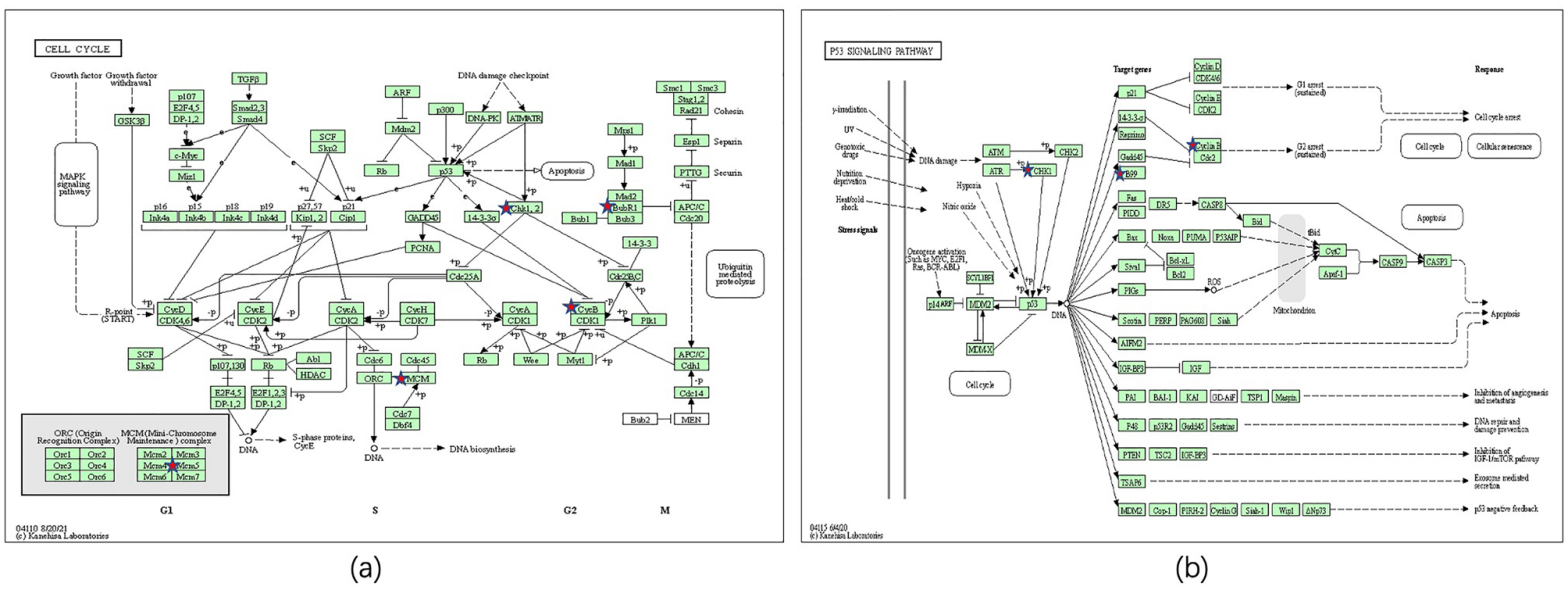
KEGG pathway. (**a**) Cell cycle pathway; (**b**) P53 pathway. Chk1 (CHEK1, checkpoint kinase 1), BubR1 (BUB1B, BUB1 mitotic checkpoint serine/threonine kinase B), Cyc B (cyclin B1, CCNB1), MCM5 (minichromosome maintenance complex component 5) and B99 (GTSE1, G2 and S-phase expressed 1).

The largest cluster in different comparisons were re-analyzed using DAVID. KEGG pathways with < 3 DEGs were excluded. Finally, two KEGG pathways, the cell cycle and p53 signaling pathways, were revealed between lesioned skin samples of AD patients and those of healthy controls (P < 0.05). Four candidate genes were illustrated in the cell cycle pathway, including cyclin B1 (CCNB1, Cyc B), checkpoint kinase 1 (CHEK1, Chk1), BUB1 mitotic checkpoint serine/threonine kinase B (BUB1B, BubR1) and minichromosome maintenance complex component 5 (MCM5) (Fig. 5a). Three candidate genes were found in the p53 signaling pathway, consisting of CCNB1(Cyclin B), CHEK1 and G2 and S-phase expressed 1 (GTSE1, B99) (Fig. 5b). No statistically significant differences in DEG-related KEGG pathways were obtained between non-lesioned skin samples of AD patients and those of healthy controls, so did lesioned and non-lesioned skin samples of AD patients.

### miRNA-gene analysis

**Figure 6:**
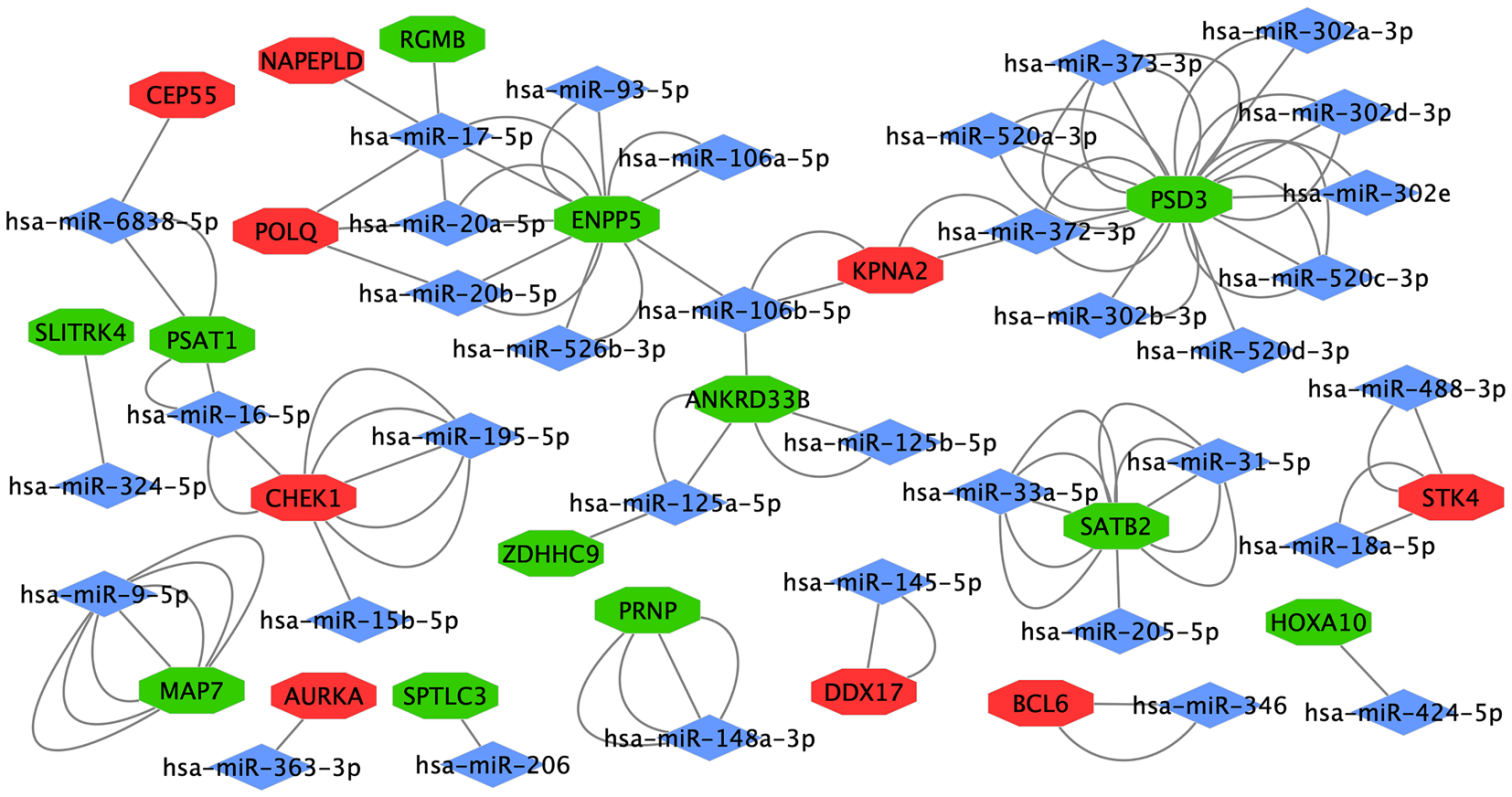
Regulatory relationships between human genes and predicted miRNA analyses as determined using miRWalk 3.0. Red indicates up-regulated genes, green down-regulated genes and blue indicates predicted miRNAs.

Regulatory relationships between human genes and predicted miRNAs were assessed using miRWalk 3.0. A total of 56 nodes (35 miRNAs, 21 genes) and 100 edges were obtained in the miRNA-target gene network. The has-miR-17-5p was targeted to 4 genes, including N-acyl phosphatidylethanolamine phospholipase D (NAPEPLD), repulsive guidance molecule b (RGMB), polymerase θ (POLQ) and ecto-nucleotide pyrophosphatase/phosphodiesterase (ENPP) 5. The has-miR-106b-5p was targeted to 3 genes, including ENPP5, ankyrin repeat domain (ANKRD) 33B and karyopherin subunit alpha (KPNA) 2. In addition, 6 miRNAs (has-miR-6838-5p, has-miR-16-5p, has-miR-20a-5p, has-miR-20b-5p, has-miR-372-3p, has-miR-125a-5p) were also identified as being individually targeted to 2 genes (Fig. 6).

## Discussion

AD is widely accepted as a common, chronic inflammatory skin disease worldwide^22^. Immune dysregulation appears to represent an important factor in the etiology of this condition^23^. Therefore, identification of novel candidate DEGs may offer new insights and highlight potential therapeutic targets for AD patients.

In our study, 225 DEGs were found in lesioned skin samples of AD patients as compared with those from healthy controls as based on the 4 GEO databases. These DEGs were predominantly enriched in inflammatory responses and cell division for BP, cytoplasm for CC and receptor binding for MF. PPI analysis revealed that 17 up-regulated and 2 down-regulated nodes were present in the largest cluster. Within the cell cycle (CCNB1, CHEK1, BUB1B, MCM5) and p53 (CCNB1, CHEK1, GTSE1) signaling pathways there were two candidate KEGG pathways. The novel predicated miRNA was has-miR-17-5p, which targets NAPEPLD, RGMB, POLQ and ENPP5.

In eukaryotes, the cell cycle composes of 4 phases, including gap 1 (G1), synthesis (S), gap 2 (G2) and mitosis (M) phase^24^. It is controlled by several tumor suppressor genes (such as p53), cyclins and cyclin-dependent kinase (CDK) complexes^25^. Phosphorylated β-catenin combines with pyruvate kinase isozyme M2 to interact with transcription factor 4. As a result, the transcription of MYC and cyclin D1 are enhance, which binds to CDK 4/6 leading to the G1 phase^26^. Cyclin-CDK then gradually phosphorylates retinoblastoma leading to the S phase, which then enables cells to replicate their genetic contents and checked for errors. The division of organelles in the G2 phase prepares cells for completion of cell division in the M phase^27^. CCNB1, a crucial member of cyclin family, is pivotal in regulating and combining with CKD1 to promote the transition from G2 to M phase^28^. CCNB1 is considered as a novel marker of mild AD, as based on bioinformatic analysis, an effect which has been corroborated from results of qRT-PCR. It may then involve in AD progression by modulation of cell proliferation, results which are consistent with the present study^21^. As for CHK1, it represents a crucial component involved with arresting of the cell cycle and repair of DNA^29^. The phosphatase cell division cycle (CDC) 25A phosphorylation and its subsequent proteasomal degradation are inhibited by CHK1, thus producing a decreased in S phase CDK2 activity. Phosphorylated CDC25B and CDC25C, as mediated by CHK1, also promotes their degradation and inhibits CDK1/cyclin B kinases activation, thus leading to G2/M arrest^30,31^. In the phase of inflammatory and proliferation in skin wound healing, CCNB1 and CHEK1 are regarded as hub genes and are vital in the cell cycle and P53 signaling pathways^32^. BUB1B is a member of the spindle assembly checkpoint (SAC) protein family, and is essential for ensuring relative chromosome segregation by suppressing anaphase onset via inhibition of APC/C activation^33^. BUB1B has also been associated with chromosomal instability, in part through p53 signaling^34^. MCM5 belongs to the MCM protein family, and is an important regulatory component in the S phase of the cell cycle. MCM5 shows poor helicase activity, prior to the onset of DNA synthesis by binding to chromatin^35^.

The P53 pathway is relative to cell division, DNA repair, cellular senescence, cell death and metabolism^36^. Recently, it was reported that large amounts of thymic stromal lymphopoietin (TSLP) are released by keratinocytes in lesioned skin samples of AD patients, an effect which leads to a considerable degree of inflammation and skin damage. As a p53-homologue, ΔNp63 expression has been shown to be elevated in keratinocytes where it inhibits the TSLP receptor complex and regulates the expressions of IL-31 and IL-33^37,38^. Suzuki’ et al. reported that p53 was increased in the IgE-mediated activation of mast cells in allergic diseases such as atopic dermatitis, while a deficiency of p53 leads to enhanced anaphylaxis responses^39^. GTSE1, which is located in the cytoplasm, regulates microtubules and cytoplasmic tubulin activity during the S phase, as well as spindle integrity and chromosomal movement during the M phase. GTSE1 also serves as a negative regulator of p53 by nucleus accumulation and bandage to p53 within the nucleus and to degrade furtherly by transporting it to the cytoplasm. An overexpression of GTSE1 results in delaying the transition process from the G2 to M phase^40^. In AD patients, CHK1/MCM5 are down-regulated while BUB1B/GTSE1 are up-regulated, as revealed from the bioinformatic analysis performed in the present study. Our current results reveal the first evidence indicating a relationships between CHK1/ BUB1B/MCM5/GTSE1 and AD.

miRNAs are short, single-stranded non-coding RNAs that modulate gene expressions by inhibiting translation of messenger RNA or promoting its degradation at the post-transcriptional level^41^. Some miRNAs, such as miR-205 and miR-451, have been demonstrated to be involved in AD^42,43^. miR-17-5p, which belongs to the miR17-92 cluster, exerts pleiotropic functions in aging, longevity, wound healing and cancer, as well as playing vital roles in cell cycle regulation, autophagy and apoptosis^44,45^. Sand’s et al. demonstrated that the miR-17–92 cluster, such as miR-17-5p, is involved in the etiology of cutaneous squamous cell carcinoma. However, no known connections between miR-17-5p and other dermatoses (such as AD) have so far been reported. The miR-17-5p in humans is has-miR-17-5p. The present results demonstrate that has-miR-17-5p is the novel predicated miRNA and is targeted at NAPEPLD, RGMB, POLQ and ENPP5, but no relationships between these genes and AD have been reported. NAPEPLD is the main synthetizing enzyme for N-acylethanolamine, which is a subgroup of bioactive lipids^46^. The DEGs were enriched in the regulation of lipid metabolic processes in BP and lipid particles in CC by our GO analysis. RGMB is an extracellular molecule and a vital partner binding to cytotoxic T-lymphocyte associated protein 4, which plays a significant role in the maintenance of immune homeostasis^47^. Moreover, the DEGs are enriched in extracellular region, extracellular space and extracellular exosome for CC, and receptor binding for MF as based on our GO analysis. POLQ is involved in DNA double-strand breaks (DSB) repairs, with deletions in POLQ resulting in deleterious consequences such as genomic rearrangements and cell death^48^. Homologous recombination represents the preferred DSB repair pathway when a sister chromatid is convenient in the S and G2 phases of the cell cycle^49^. The present results demonstrate that the DEGs are enriched in cell division for BP on the based of the GO analysis and the cell cycle as based on the KEGG pathway. ENPP5 contains 3 Zn^2+^ ions in the catalytic site, which interact with the 2′ and 3′ oxygens of ribose substrates. Moreover, the tyrosine (Tyr73), in the nucleotide-binding slot in ENPP5, eliminates hydroxyl groups that may conflict with nucleotide substrates, thus enabling nucleotriphosphatase hydrolysis^50,51^. As the GO analysis results of our study illustrate that the DEGs obtained are enriched in iron ion binding for MF, we hypothesize that has-miR-17-5p might be related to the regulation of immune responses by increasing the expressions of NAPEPLD and POLQ and decreasing the expressions of RGMB and ENPP5.

Prior to our current report, a total of 6 studies have been published on DEGs in AD, as based on bioinformatic analysis. Only 1 or 2 GEO datasets were included in Peng et al. (GSE63741, GSE124700)^52^, Li et al. (GSE32924, GSE31408)^53^, Zhang et al. (GSE6012)^54^, Yin et al. (GSE75890)^21^ and Ding et al. (GSE32924)^55^. Although 4 GEO datasets were included in Wang et al. (GSE32924, GSE36842, GSE58558, and GSE107361), theses samples were limited to pediatric AD patients. In the present study, we present data from 571 adult skin samples within 9 GEO datasets as analyzed using bioinformatic analysis. The number of the databases and samples contained in our study represents a substantial increase over that of previous reports, thus providing a more comprehensive and reliable assessment of this issue.

In conclusion, the results of our study provide some new insights into the dysfunctions of inflammation and immune responses as associated with AD. Such information is critical for the identification and development of potential therapeutic targets to treat AD. However, some limitations in our study require consideration. As the DEGs identified were confined to the GEO dataset, data available in databases other than GEO were not included in our analysis. In addition, corroborative findings, as achieved with some experiments in vitro or in vivo, will be required to verify the results presented in this study.

## Data Availability

The datasets generated and/or analyzed during the current study are available in the GEO repository (GSE16161, GSE32924, GSE59294, GSE99802, GSE107361, GSE120899, GSE130588, GSE133385 and GSE133477).
